# Development of Real-Time PCR Array for Simultaneous Detection of Eight Human Blood-Borne Viral Pathogens

**DOI:** 10.1371/journal.pone.0043246

**Published:** 2012-08-17

**Authors:** Natalia Pripuzova, Richard Wang, Shien Tsai, Bingjie Li, Guo-Chiuan Hung, Roger G. Ptak, Shyh-Ching Lo

**Affiliations:** 1 Tissue Microbiology Laboratory, Division of Cellular and Gene Therapies, Office of Cellular, Tissue and Gene Therapy, Center for Biologics Evaluation and Research, Food and Drug Administration, Bethesda, Maryland, United States of America; 2 Department of Transfusion Medicine, The Warren Grant Magnuson Clinical Center, National Institutes of Health, Bethesda, Maryland, United States of America; 3 Department of Infectious Disease Research, Southern Research Institute, Frederick, Maryland, United States of America; Institut Pasteur of Shanghai, Chinese Academy of Sciences, China

## Abstract

**Background:**

Real-time PCR array for rapid detection of multiple viral pathogens should be highly useful in cases where the sample volume and the time of testing are limited, i.e. in the eligibility testing of tissue and organ donors.

**Findings:**

We developed a real-time PCR array capable of simultaneously detecting eight human viral pathogens: human immunodeficiency virus types 1 and 2 (HIV-1 and -2), hepatitis B virus (HBV), hepatitis C virus (HCV), human T-cell leukemia virus-1 and -2 (HTLV-1 and -2), vaccinia virus (VACV) and West Nile virus (WNV). One hundred twenty (120) primers were designed using a combination of bioinformatics approaches, and, after experimental testing, 24 primer sets targeting eight viral pathogens were selected to set up the array with SYBR Green chemistry. The specificity and sensitivity of the virus-specific primer sets selected for the array were evaluated using analytical panels with known amounts of viruses spiked into human plasma. The array detected: 10 genome equivalents (geq)/ml of HIV-2 and HCV, 50 geq of HIV-1 (subtype B), HBV (genotype A) and WNV. It detected 100–1,000 geq/ml of plasma of HIV-1 subtypes (A – G), group N and CRF (AE and AG) isolates. Further evaluation with a panel consisting of 28 HIV-1 and HIV-2 clinical isolates revealed no cross-reactivity of HIV-1 or HIV-2 specific primers with another type of HIV. All 28 viral isolates were identified with specific primer sets targeting the most conserved genome areas. The PCR array correctly identified viral infections in a panel of 17 previously quantified clinical plasma samples positive for HIV-1, HCV or HBV at as low as several geq per PCR reaction.

**Conclusions:**

The viral array described here demonstrated adequate performance in the testing of donors’ clinical samples. Further improvement in its sensitivity for the broad spectrum of HIV-1 subtypes is under development.

## Introduction

Rapid progress and improvement in molecular technologies have allowed researchers to switch from the traditional approaches of virus detection in clinical samples to multiplexing for simultaneous detection of multiple pathogens in a single assay. A number of different PCR based assays for detection and discovery of multiple pathogens have been developed [1]–[4]. Detection microarrays are proven to be useful in the identification and discovery of viruses homologous to known species. They have been used to guide the selection of samples for further analysis by sequencing [1]–[3], [5]. However, microarrays based on nucleic acid hybridization are too complex in design and performance for the routine donors testing, and exhibit a comparatively low sensitivity of detection, usually around 100−1,000 genome copies of target virus per analyzed sample [1]–[2]. Several PCR based assays coupled with oligonucleotide microarray technology (so called re-sequencing arrays) have been designed to allow simultaneous detection or genotyping of a target group of viruses, such as some critical blood-borne pathogens (3 viruses) [6], respiratory viruses (16–21 viruses) [7]–[8], and respiratory adenoviruses (6 different serotypes) [9]. Such PCR based approach allows increasing the sensitivity of detection down to 10–100 copies of the target RNA or DNA in a sample.

PCR multiplexing should be highly useful when both the volume of the samples and the time of testing are critical, as in the donor eligibility (DE) testing for tissue or organ transplantation [10]. Current regulation requires that DE testing be performed using assays approved and licensed by the U.S. Food and Drug Administration (FDA). However, the automated assay systems that are designed to screen large numbers of samples, without the strict limitation of sample volumes, may not be completely suitable or ideal for the needs of DE testing for tissue or organ transplantation.

The main goal of the study presented here was to evaluate the feasibility of developing a sensitive and specific assay for rapid detection and identification of a group of target viral pathogens. The following viral pathogens were included in our array: human immunodeficiency virus types 1 and 2 (HIV-1 and HIV-2), human T-cell leukemia virus-1 and -2 (HTLV-1 and HTLV-2), hepatitis C virus (HCV) and West Nile virus (WNV), all with single-stranded RNA genome; vaccinia virus (VACV) and hepatitis B virus (HBV), both with double-stranded DNA genome, HBV also has single-stranded RNA stage. Some of the listed viruses are included to the required DE testing for tissue transplantation. Besides, historically, some of the targeted viruses have been found to be allograft-transmitted to recipients [11]–[12]. In the present study, a real-time PCR array with SYBR-Green chemistry targeting these eight viral pathogens listed above was developed and evaluated with analytical and clinical panels. The array demonstrated acceptable performance in the testing with both analytical panels and donors’ clinical samples.

## Materials and Methods

### Panel of Clinical Blood Samples

The research study conducted at FDA using previously frozen blood samples was reviewed by Department of Health and Human Services, Food and Drug Administration, Research Involving Human Subjects Committee (RISHC Protocol #10-008B entitled “Detection of Infectious Agents in Previously frozen blood Samples from Patients with Various Illnesses and Healthy Blood Donors”). The 17 clinical plasma samples positive for HBV, HCV or HIV-1 used in this study were existing clinical diagnostic samples kept in NIH Blood Bank. Information of these left over samples had been recorded in such a manner that subjects can not be identified, directly or through identifiers. The written informed consent from the participants was waived under 45 CFR 46.101 (b) (4). The six plasma samples positive for HIV-1 were estimated to contain 50 to ∼90,000 genome copy numbers per ml; six plasma samples positive for HCV were estimated to contain 780 to ∼123,000 genome copy number/ml and five plasma samples positive for HBV were estimated to contain ∼150 to 16,000 copy number/ml. The copy numbers of viruses in these samples were provided by the NIH Blood Bank. No information about the subtypes or genotypes of these viruses was available. The amount of each clinical sample was sufficient to be tested only once by the PCR array in the study. All positive PCR products obtained in the testing using the PCR array were confirmed for validity by sequencing in the Facility for Biotechnology Resources of FDA/CBER.

### Primers Design

We used the “Insignia” program (http://insignia.cbcb.umd.edu/query.php), a bioinformatics on line tool developed in the Center for Bioinformatics and Computational Biology, University of Maryland [13] to choose a specific DNA or RNA “signature” (a sequence, with customized length and G/C content) for targeted viruses. Comparative sequence analysis of the complete genomes was performed using mVISTA (http://genome.lbl.gov/vista/mvista/submit.shtml). Multiple nucleotide sequence alignments (NSAs) were then created to visualize the most conserved genome areas using MEGA4 (http://www.megasoftware.net).

Specific criteria for the primers and amplicon selection for the SYBR Green based PCR array were: 1) the same range of annealing temperature (T) -57–60°C - for all primers, 2) high G/C content for primers, allowing higher specificity of annealing, and 3) an amplicon size in the range of 100–200 b.p. in order to have a high PCR amplification efficiency and to sufficiently distinguish the products from primer dimers based on melting T peak (Tm). All primers were checked for potential dimer formation using “Primer Express” software (version 3.0, Applied Biosystems). After design, all primers were again checked using the National Center for Biotechnology Information (NCBI) Basic Local Alignment Search Tool (BLAST) (http://blast.ncbi.nlm.nih.gov/Blast.cgi) to avoid any cross-reactivity with other species. Newly designed and previously published primer sets adapted for the final version of the real-time PCR array are listed in Table 1. In addition, primers specific for the human beta-globin gene were included in the array as an internal control for the quality of DNA/RNA preparation as well as for estimation of viral copy number per host cell if needed (the last row of Table 1).

**Table 1:** List of primers selected for the real-time PCR array based on their specificity and sensitivity with in-house analytical panels.

Virus	Gene targeted	Primer name^a^	Primer sequence	Variants′ coverage^b^	Analytical sensitivity (copies/PCR)^c^	Design^d^
HIV-1	*gag*	NP3	ATAATCCACCTATCCCAGTAGGAGAAAT	partial group M	10	[28]
		NP4	TTTGGTCCTTGTCTTATGTCCAGAATG			
	*pol*	NP51	GCAGCATAGAACAAAAATAGAGGAGC	partial group M	5	Insignia
		NP52	GGGTAAATCTGACTTGCCCAATTC			
	*pol*	NP170	GA**R**ACCAA**R**AATGATAGG**R**GGAATTG	group M	10	CGA
		NP171	CCAATTATGTTGACAGGKGT**R**GG			
	*pol*	NP175	G**R**GAAAGAATA**R**TAGACATAATAGC	group M	10	CGA
		NP174	CTACYGCCCCTT**Y**ACCTTTCC			
HIV-2	*gag*	NP84	GTAGACCAACAGCACCACCTAG	HIV-2/SIV	10	Ins./CGA
		NP85	CTGGCACTACTTCTGCCCCG			
	*gag*	NP86	GGGCAGAACAAACAGACCCAGC	HIV-2	2.5	Ins./CGA
		NP87	GGCAGGCGGTTAGCATCTCTTC			
	*env*	NP76	CTCGCCTCTTGAYCGG**R**CTATAC	HIV-2	5	Ins./CGA
		NP77	CCCGCAAGAGTCTCTCTCGTAG			
HTLV-1	*env*	NP88	CTCCTCCCCCTGTCATAACTC	HTLV-1 Sub. A/C	5	Ins./CGA
		NP89	GAGACAAGCCAGACYGCCAC			
	*env*	NP47	G**R**TTACCGGCYCCATGTCCC	HTLV-1 Sub. A	10	Ins./CGA
		NP48	C**R**GCACTGTTCTTGTAATGCTTTGC			
	*pol*	NP90	GC**RR**CAGGCCCTGTCACAG	HTLV-1 Sub. A/C	10	CGA
		NP91	GTGGTGCCAGTGAGGGTYAGC			
HTLV-2	*pol*	NP63	CGGCCTTACCAATCAGCGGTG	HTLV-2 Sub. A	5	Ins./CGA
		NP64	G**R**GTTCGCTGGCGTCTGGTC			
	*env*	NP65	CTCCATYCCAACCCTYCCCTTG	HTLV-2 Sub. A	10	Ins./CGA
		NP66	GC**R**CGGCGGCGTCTTGTCG			
	*pol*	NP67	GGCATCCTCATTCACCACATGGG	HTLV-2 Sub. A/B	10	Ins./CGA
		NP68	CAGGGGGCCGTGTCAAGCAC			
HBV	S gene	NP11	GT**R**TCTGCGGCGTTTTATCA	Gen. A–H excl. some D	10	[29], [30]
		NP97	GACA**M**ACGGGCAACATACC			
	Core	NP94	GACCACCAAATGCCCCTATC	Gen. A–H	10	Insignia
		NP100	ATTGAGATCTTCTGCGACGCGG			
	S gene	NP11-m	GT**D**TCTGCGGCGTTTTATCA	Gen. A–H	10	CGA
		NP97-m	GACA**N**ACGGGCAACATACC			
HCV	5′UTR	NP13	CAGAAAGCGTCTAGCCATGGCGT	Most of Gen. 1–6	2.5	[6]
		NP14	ACTCGCAAGCACCCTATCAGGCA			
	5′UTR	NP13-m	C**R**GAAAGCGYCTAGCCATGGCGT	Gen. 1–6	2.5	CGA
		NP14	ACTCGCAAGCACCCTATCAGGCA			
WNV	E protein	NP21	TCAGCGATCTCTCCACCAAAG	WNV	10	[31]
		NP22	GGGTCAGCACGTTTGTCATTG			
	NS5	NP176	CCCCTACATGCCGAAAGTCATAGA	WNV	10	CGA
		NP177	CACCCAATACATCTCGTGCGTGG			
	NS5	NP178	TGAAGACCACTGGCTTGGAAG	WNV	10	CGA
		NP179	CCAGCCTGCTGTGTCATCAG			
VACV	HA*	NP102	GGACAATCTGACCATCCATYGC	VACV	10	Insignia
		NP103	CCTCGTCTTCTTCTACATCA**R**CC			
	ORFA9L*	NP104	CGGATCCAAATGCTGTCTG**Y**G	VACV	10	Insignia
		NP105	GG**M**ATTYACTTCGTATAGCGG			
Human	ß-globin	NP23	ACACAACTGTGTTCACTAGC	human/primate	5	[32]
		NP24	CAACTTCATCCACGTTTCACC			

a - to simplify the process of evaluation we used our primer names with sequential numbers, however some of the primers have been designed previously with their original names in the articles listed in the last column of this table;

b - variants’ coverage is based on multiple nucleotide sequence alignments performed “in house”;

c - analytical sensitivity here is based on testing with in-house DNA standards;

d – primers were selected from sited studies or designed “in-house” using DNA/RNA signatures generator “Insignia” (Ins.) or comparative genome analysis (CGA); -m – modified; Sub. – subtypes; Gen. – genotypes; Excl. – excluding; M (A or C), R (A or G), K (G or T), Y (C or T), N (A or G or T or C), D (A or G or T); HA – Haemagglutinin, *VACV genome annotation is according to reference [33].

### In-house Positive Standards

Total cellular DNA of the chronically infected cell cultures (for HIV-1, HIV-2; HTLV-1, HTLV-2 and VACV), viral genome cDNA copy spiked into human DNA (for HCV and WNV), and DNA isolated from human plasma of the infected individual (for HBV) were used as the positive templates in the initial testing and are listed in the last column of Table S1. DNA or RNA panels were created by cloning of specific synthetic templates for each virus into the pGEM-T-Easy vector (Promega) by TA cloning, following by *in vitro* transcription to obtain RNA standards for HCV and WNV. All created plasmids are listed in Table S1. Nucleotide numbers in Table S1 refer to the location of the partial viral genome cloned into pGEM-T-Easy vector according to the following complete genome sequences available in GenBank: HTLV-1 - L03562.2, HTLV-2 - M10060.1, HIV-1 - K02083.1, HIV-2 - J03654.1, HBV - AF462041.1, HCV - AF271632.1, VACV - AY243312.1, WNV - HQ596519.1. To establish the real-time PCR standard curve the copy number was calculated for each plasmid carrying one copy of the specific viral gene. The size of each plasmid (X b.p.) was used to determine the molecular weight in Daltons (g/mol): W (g/mol) =  X b.p. (330 Da × 2). The copy number of the target viral gene (molecules/µl) was determined from the plasmid concentration (C_DNA_) and the molecular weight of each plasmid molecule:

Copy number  = C_DNA_ (ng/µl) × 6.02 × 10^23^(Avogadro’s number)/W.

Knowing the number of plasmid molecules with the target viral gene in a µl, a series of dilutions was made to generate a PCR standard curve.

The developed analytical standards were used to calculate the intra and inter-assay reproducibility of quantification for each virus-specific primer set. Mean C(t) values, standard deviation (SD) and coefficient of variation (CV) were calculated from the data obtained in three replicates of each standard dilution for the intra-assay reproducibility, and in three real-time PCR assays consisted of three replicates each (nine total) for the inter-assay reproducibility. CV was calculated as SD/Mean C (t) * 100%.

### Preparation of Viral RNA/DNA for PCR Array Analysis

0.5–1 ml of human plasma was used for the total viral RNA/DNA extraction using “QIAamp Viral RNA Mini-Kit” (*Qiagen*) and tRNA (*SIGMA*) was used as a carrier RNA during the preparation. After the final elution step RNA/DNA in 160 µl of buffer AVE was precipitated with 100% ethanol and 3 mM NaCl at −20°C overnight. An RNA/DNA pellet was washed with 70% ethanol, dissolved in 10 µl of DEPC-treated water and then immediately used for cDNA synthesis with SuperScript II RT (*Invitrogen*) and random hexamers (*Invitrogen*) in a total reaction volume of 20 µl. The volume of cDNA/DNA sample was then adjusted up to 30 µl with DEPC-treated water and the whole volume (1.25 µl per each of 24 primer sets) was immediately used for PCR array testing.

### PCR

PCR was performed using “Bio-Rad CFX96 Real-Time System” with “Power SYBR Green PCR master mix” (Applied Biosystems). One reaction (25 µl total) contained: 12.5 µl of PCR master mix, 0.5 µM of each primer and 1.25 µl of DNA/cDNA template. In the single virus testing (sections 3 and 4 of the “Results”) we used 2.5 µl of DNA/cDNA template from 20 µl of sample after cDNA synthesis. “Universal” PCR conditions for all primer sets included to the array were: 95°C for 8 min (one cycle), then 50 cycles of: denaturation at 95°C for 15 s, annealing and extension at 60°C for 1 min, followed by melting curve read from 65°C to 95°C with increment 0.2°C for 5 s. Real-time PCR data were downloaded in 96-well plate format from “Bio-Rad CFX Manager 2.1” to MS Excel and analyzed manually.

### Statistical Analysis for Determination of Threshold Cycle (C(t)) Cut off for PCR Array

Two types of samples served as the background control for determination of the C(t) cut off. The 1^st^ type of negative control was 50 ng of human cellular DNA. The 2^nd^ type of negative control was negative donors’ plasma. Data were collected in separate experiments from 8 human cellular DNA controls and 3 negative donors’ plasma. To standardized the C(t) cut off for all primers the threshold was set at the PCR machine default setting. Based on a false positive rate of less than 5% the following method [14] was used to estimate the C(t) cut off from the range of C(t) obtained with negative samples for all 24 primer sets in the array:

1. Calculate the margin of error of the confidence interval (CI), W = t* × SD/√n, where: n – number of obtained C(t) values, SD– standard deviation, df = n−1, and t* (for 95% confidence) is a “critical value of the T distribution” [14].
2. One side CI covers this range: M–W, where M is sample mean.
3. Positives have C(t)<(M–W) and negatives have C(t)≥(M – W).

The C(t) cut off calculated from the range (n = 50) of the 1^st^ type of negative control data was C(t)≤41.03. The C(t) cut off calculated from the range (n = 50) of the 2^nd^ type of negative control data was C(t)≤42.7. Even some overlap between the C(t) measurements of truly positive and truly negative samples was detected in PCR array data, the Tm parameter was used to define if the obtained PCR product is specific by comparison to the expected Tm peak range.

### Analytical Plasma Panels from FDA/CBER

The analytical sensitivity of each primer set was determined in the single virus testing using FDA/CBER panels (kindly provided by Dr. Stephen Kerby, FDA/CBER) consisting of various amounts of the viruses (0–1,000 genome copies/ml) spiked into the “normal” human plasma. Panels for the following viruses were used in testing: HIV-1 (three different panels), HIV-2, HBV (based on genotype A), HCV (genotype 1b), and WNV (based on strain HU2002). These panels have been used for testing of commercially licensed NAT assays and their development was described previously [15]. Three separate HIV-1 RNA panels were used for testing. The first consisted of various amounts of an HIV-1 group M subtype B isolate: 0, 5, 10, 25, 50, 100, and 500 copies/ml. The second consisted of 25 samples representing various concentrations of HIV-1 groups O and N, and group M subtypes A, C, D, E, F and G: 10, 100 and 1,000 copies/ml for each virus. The third panel consisted of HIV-1 circulating recombinant forms (CRFs) AE and AG: 100, 1,000, and 10,000 copies/ml for each CRF.

### HIV-1 and HIV-2 Diversity Panel

The HIV-1 and HIV-2 diversity panel developed at Southern Research Institute (Frederick, MD) consisted of different clinical isolates of HIV-1 and HIV-2. Twenty-eight (28) different isolates: three from each subtype from A to G of HIV-1 group M, three from HIV-1 group O, one from HIV-1 group N and three different isolates of HIV-2 were included in this panel. All isolates were obtained from the NIH AIDS Research and Reference Reagent Program (ARRRP), Division of AIDS, NIAID, NIH as follows (catalog number; HIV-1 group, and Envelope subtype, when appropriate, indicated in parentheses): HIV-1 isolates 92RW009 (#1747; M, A), 92RW020 (#1749; M, A), 92UG037 (#1743; M, A), 92BR025 (#1777; M, C), 92UG001 (#1647; M, D), 92UG035 (#1742; M, D), 92TH006 (#1654; M, EA), 93TH073 (#2390; M, E), 93BR019 (#2314; M, F), 93BR020 (#2329; M, F), and 93BR029 (#2338; M, F) from the UNAIDS Network for HIV Isolation and Characterization [16]; HIV-1 isolate JR-CSF (#394; M, B) from Dr. Irvin Chen [17]–[18]; HIV-1 isolate 92US660 (#1722; M, B) from The Multi-center AIDS Cohort Study; HIV-1 isolate 92HT599 (#3301; M, B) from Dr. Neal Halsey; HIV-1 isolate 93IN101 (#2900; M, C) from Dr. Robert Bollinger; HIV-1 isolate 93MW959 (#2915; M, C) from Dr. Paolo Miotti; HIV-1 A07412M1 (#11261; M, D) from Dr. Victoria Polonis [19]; HIV-1 isolate CMU08 (#3025; M, E) from Dr. Kenrad; HIV-1 isolates JV1083 (#3191; M, G) and G3 (#3187; M, G) from Dr. Alash’le Abimiku [20]; HIV-1 isolate RU132 (#3509; M, G) from Dr. A. Bobkov and Dr. Jonathon Weber; HIV-1 isolates YBF30 (#4145; N), BCF01 (#3333; O), BCF02 (#3334; O), and BCF03 (#3335; O) from Drs. Sentob Saragosti, Françoise Brun-Vézinet, and François Simon [21]–[22]; HIV-2 isolate CBL-20/H9 (#600) from Dr. Robin Weiss [23]; HIV-2 isolate CDC310319 (#3930) from Dr. Stefan Wiktor and Dr. Mark Rayfield [24]; and HIV-2 isolate MVP-15132 (#803) from Dr. Lutz Gürtler and Dr. Friedrich Deinhardt [25]–[26]. HIV-1 groups and subtypes are based on designations reported by the NIH ARRRP. Low passage virus stocks were prepared and median tissue culture infective dose (TCID_50_)/ml of virus containing supernatant determined in fresh human PBMCs isolated as previously described [27]. Infectious unit (IU) of the virus stocks in TCID_50_s were titrated for each isolate and the number of IU used for viral RNA isolation and PCR was calculated based on the dilution factor (1∶100) and ranged from ∼10 to 2,500 or 1 to 3.4 log_10_ TCID_50_ per PCR reaction.

## Results

### 1. Design of a Real-time PCR Viral Array using Combined Bioinformatics Approaches

PCR approach based on SYBR Green chemistry, allowing simultaneous detection of multiple targets, was chosen to be applied for the array performance. Virus-specific primer sets targeting at least three different genomic sites for each viral pathogen were designed for the real-time PCR array. We used the “Insignia” program, a bioinformatics tool that helps to choose a specific DNA or RNA “signature” for different bacteria and viral pathogens that are included in the pre-built “Insignia” database [13]. Sequences of the highly conserved regions, such as coding viral polymerase or structural proteins were selected to design the candidate primers. In addition, we performed nucleotide sequence alignments (NSA) of the complete viral genomes and of the most conserved genome areas for different subtypes/genotypes or different isolates of all targeted viruses. Some previously published primers designed for PCR detection of the target viruses were also adapted and evaluated. Overall, a total of 120 primers were initially designed using specific criteria for the current real-time PCR array (see Materials and Methods) to cover the eight targeted viruses: HIV-1, HIV-2, HBV, HCV, HTLV-1, HTLV -2, WNV, and VACV. The primers were designed and tested for their effectiveness and specificity of amplifying the respective target under uniform PCR conditions (using the same annealing temperature for all primers).

### 2. Experimental Testing and Selection of the Virus Specific Primer Sets for the Real-time PCR Array

Each candidate primer pair was first tested for its specificity and sensitivity of PCR amplification. This was assessed in a real-time PCR cross-testing against human PBMC DNA (50 ng/PCR reaction) from a healthy donor, DNA from human cell cultures infected by various target viruses, or human DNA spiked with a known amount of genome copies of various viruses. It was important to ensure that the selected primers targeting a specific virus would not non-specifically amplify any DNA in a sample. The melting temperature (Tm) peak of the product amplified from the target virus should be clearly differentiable from the Tm peak of primer dimers or any non-specific products produced in PCR. DNA or RNA panels were created from the cloned synthetic templates (listed in Table S1) by spiking of 2–10^4^ genome copies of each virus into 50 ng of human background DNA. The sensitivity of each candidate primer set for each target virus was assessed using these panels.

Example of the experimental testing of HIV-1 specific primer set targeting *gag* gene (NP3/4) for its sensitivity with DNA analytical standards is shown in Figure S1. Serial dilutions of a plasmid DNA corresponding to 5–10^4^ HIV-1 genome copies spiked into 50 ng of normal human DNA, HIV-1 infected cells (H9/IIIB) (“positive” control DNA) and uninfected human PBMC (“negative” control DNA) are used in the experiment. Only a single melting peak Tm = 72.5°C corresponding to HIV-1 specific product was observed and no unspecific amplification was registered. Figure S1B revealed a standard curve showing the correlation between copy number of the target gene and Ct values with a slope  = −3.77. The limit of sensitivity was determined in this assay to be 10 viral genome copies/PCR.

After evaluation, a total of 24 primer pairs targeting the eight different viruses were chosen for the real-time PCR array based on their specificity and sensitivity (Table 1). Among them, five of the primer sets were previously published and the other 19 primer sets were originally designed in the current study. Table 1 shows the sequences of the previously published primer sets selected for the real-time PCR array with the reference to the original source. Analytical sensitivity expressed in genome copy/PCR for each primer set (Table 1) was estimated using DNA/RNA analytical panels, as described above. Coverage of variants (i.e., different subtypes or genotypes) for each virus (Table 1) was estimated using the NSAs. Degenerative nucleotides were introduced into some of the primer sets based on the NSAs performed in-house to obtain a broader coverage. The Tm peak range of the product stated in Table 1 for each primer set was established during further testing of selected primers with analytical and clinical panels.

In addition, the intra and inter-assay reproducibility of quantification for all primer sets was evaluated using three replicates of each standard dilution (of DNA or RNA analytical standards) in each of three real-time PCR assay runs. The coefficient of variation (CV) for the C (t) values was ≤3.3% and ≤6.7% for intra- and inter-assay, respectively. All the data depicting mean C (t), standard deviation (SD), and CV for each primer set selected for the real-time PCR array with each standard concentration are shown in Table S2.

### 3. Validation of Assay Sensitivity for Each Selected Primer Set with Analytical Panels Developed for Licensed Nuclear Acid Amplification Testing (NAT) Assays

To further evaluate the sensitivity of the selected primers, we used FDA/CBER panels (kindly provided by Dr. Stephen Kerby, FDA), consisting of various amounts of viruses spiked into “normal” human plasma, that are specifically developed and used for the evaluation of commercially licensed NAT assays. Table 2 summarizes the testing results for the selected primers against the target viruses. One out of four primer sets targeting HIV-1, NP3/4, could detect the subtype B HIV-1 RNA at the concentration of 50 copies/ml of human plasma. Two other HIV-1 specific primer sets (NP51/52 and NP170/171) detected the HIV-1 RNA at 100 copies/ml of plasma. The 4th primer set, NP175/174, (targeting the conserved *pol* region and containing degenerative nucleotides to support broader variant coverage) could detect the virus only at 500 viral genome copies/ml of plasma. HIV-2 RNA was detected with primer set NP86/87 at the concentration of 10 copies/ml and with the primer set NP76/77 at the concentration of 50 copies/ml. Another primer set (NP84/85) detected HIV-2 at 100 copies/ml. HBV (genotype A) DNA was detected with the primer set NP11/97 at 50 copies/ml and with NP94/100 at 100 copies/ml. The third HBV specific primer set (NP11/97-mod) detected HBV at 500 copies/ml of plasma. Both HCV specific primer sets targeting the viral 5′NTR detected HCV RNA at the concentration of 10 copies/ml of plasma. Two of the WNV-specific primer sets (NP21/22, targeting E protein gene, and NP176/177, targeting NS5) detected WNV at 50 copies/ml and the third primer set (NP178/179, also targeting NS5) gave a positive signal at 100 copies/ml of plasma.

**Table 2:** The results of sensitivity testing of the real-time PCR array primer sets specific for HIV-1, HIV-2, HBV, HCV, and WNV the with FDA/CBER analytical plasma panels.

Virus (isolate) and panel member number	Copy number/ml of plasma^1^	Result
HIV-1 RNA (subtype B)	(copy number/PCR)	4 primer sets
1901	**0**	−/−/−/−
1902	**10 (1.25)**	−/−/−/−
1903	**50 (6.25)**	**+/−/−/−**
1904	**100 (12.5)**	**+/+/+/−**
1905	**500 (62.5)**	**+/+/+/+** 2
1906	**100 (12.5)**	**+/+/+/−**
1907	**25 (3.1)**	−/−/−/−
1908	**5 (0.6)**	−/−/−/−
**HIV-2 RNA**		**3 primer sets**
1	**0**	−/−/−
2	**5 (0.6)**	−/−/−
3	**10 (1.25)**	−/+/−
4	**50 (6.25)**	**−/+/+**
5	**100 (12.5)**	**+/+/+**
**HBV DNA (genotype A, serotype adw2)**		**3 primer sets**
1	**0**	−/−/−
2	**10 (1.25)**	−/−/−
3	**100 (12.5)**	**+/+/−**
4	**50 (6.25)**	**+/−/−**
5	**500 (62.5)**	**+/+/+**
**HCV RNA (genotype 1b)**		**2 primer sets**
2001	**0**	−/−
2002	**5 (0.6)**	−/−
2003	**10 (1.25)**	**+/+**
2004	**50 (6.25)**	**+/+**
2005	**100 (12.5)**	**+/+**
2006	**500 (62.5)**	**+/+**
**WNV RNA (strain HU2002)**		**3 primer sets**
1	**0**	−/−/−
2	**5 (0.6)**	−/−/−
3	**10 (1.25)**	−/−/−
4	**50 (6.25)**	+/+/−
5	**100 (12.5)**	+/+/+
6	**500 (62.5)**	+/+/+
7	**1000 (125)**	**+/+/+**

1 – RNA/DNA, recovered from 1 ml of plasma spiked with a known amount of virus, was converted to cDNA and divided into eight PCR reactions (the dilution factor was equal to 8). Thus, two to three PCR repeats in the same run were performed for each primer set;

2 - + means the virus was detected in 2 out of 2 or 3 out of 3 PCR repeats for each primer set; four plus (**+/+/+/+**) means that the virus was detected with all four primer sets. The order of the primers in testing was the following: four primer sets targeting HIV-1: NP3/4, NP51/52, NP170/171 and NP175/174; three primer sets targeting HIV-2: NP84/85, NP86/87 and NP76/77; three primer sets targeting HBV: NP11/97, NP94/100 and NP11/97-mod; two primer sets targeting HCV: NP13/14 and NP13/14-mod; three primer sets targeting WNV: NP 21/22, NP176/177 and NP178/179.

### 4. Sensitivity and Specificity of Amplifying different HIV-1 Subtypes and HIV-2 Isolates by the Selected HIV-specific Primer Sets

The four HIV-1 specific primer sets were further evaluated by testing them against another FDA/CBER analytical panel containing a broad spectrum of HIV-1 subtypes. As summarized in Table 3, the HIV-1 subtype F (group M) and group O isolates in the panel could not be successfully amplified by any of the four selected primer sets (detection limit is >1,000 RNA copy/ml of plasma). All other subtypes and CRFs of group M, and the group N isolate could be amplified with at least one out of the four primer sets at 50 to 1,000 RNA copies/ml of plasma (Table 3). The Tm peaks of the PCR products amplified from different subtypes of HIV-1 varied within 1.5°C (Table 3). All of the four selected HIV-1 specific primer sets amplified HIV-1 subtype B (group M) with the highest sensitivity (50–500 copies/ml of plasma).

**Table 3:** Sensitivity of four HIV-1 specific primer sets selected for the real-time PCR array in testing with FDA/CBER analytical HIV-1 broad spectrum panel.

Group/Subtype	NP3/4 (*gag*)		NP51/52 (*pol*)		NP170/171 (*pol*)		NP175/174 (*pol*)	
Group M	Detection limit^1^	Tm	Detection limit^1^	Tm	Detection limit^1^	Tm	Detection limit^1^	Tm
**A**	>1,000	N/P	>1,000	N/P	**1,000 (125)**	***74.6***	>1,000	N/P
**B**	**50 (6.25)**	***72.8–73.4***	**100 (12.5)**	***77.8***	**100 (12.5)**	***73.8***	**500 (62.5)**	***73.6–73.8/76.6–76.8*** 2
**C**	>1,000	N/P	>1,000	N/P	**1,000 (125)**	***73.4–73.6***	>1,000	N/P
**D**	>1,000	N/P	>1,000	N/P	**100 (12.5)**	***74.6–74.8***	>1,000	N/P
**E**	>1,000	N/P	>1,000	N/P	**1,000 (125)**	***73.2***	>1,000	N/P
**F**	>1,000	N/P	>1,000	N/P	>1,000	N/P	>1,000	N/P
**G**	>1,000	N/P	>1,000	N/P	**1,000 (125)**	***73.8***	>1,000	N/P
**Group O**	>1,000	N/P	>1,000	N/P	>1,000	N/P	>1,000	N/P
**Group N**	>1,000	N/P	>1,000	N/P	>1,000	N/P	**1,000 (125)**	***74.8/76.4*** 2
**CRF AE**	**10,000 (1,250)**	***72.8***	>10,000	N/P	**100 (12.5)**	***74.2***	**1,000 (125)**	***74.8/76.4*** 2
**CRF AG**	**10,000 (1,250)**	***72.6***	>10,000	N/P	**1,000 (125)**	***73.4–73.8***	**1,000 (125)**	***73.4/76.6*** 2

FDA/CBER HIV-1 RNA panel consisted of different concentrations of HIV-1 group M isolates (subtypes **A–G**, 1 isolate per subtype), one group O, one group N and two CRF (circulating recombinant form) isolates.

1 Detection limit (copy number/ml of plasma) was evaluated using FDA/CBER analytical panel, containing pre-set copy number of HIV-1 spiked into 1 ml of “normal” human plasma. RNA from 1 ml of plasma was converted to cDNA and divided into eight PCR reactions; two PCR repeats were performed for each primer set. The estimated copy number per PCR reaction is shown in parentheses.

2 Double Tm peak was always registered for this primer set with any tested HIV-1 isolates. N/P – no PCR product was detected.

**Figure 1:**
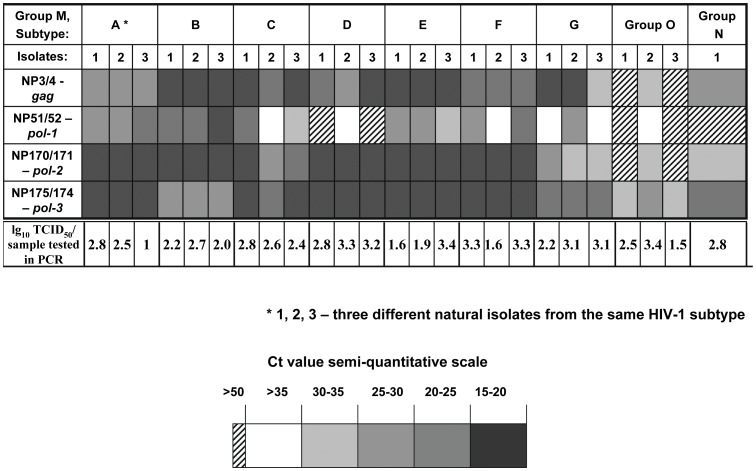
Semi-quantitative evaluation of four primer sets specific for HIV-1 with HIV-1 diversity panel. Total 25 HIV-1 clinical isolates from group M, O and N with three different isolates (shown as 1, 2, 3; only one group N isolate) from each HIV-1 group/subtype were used in testing. Infectious unit (IU) of the virus stocks in TCID_50_s were titrated for each isolate; the number of IU used for viral RNA isolation and PCR was calculated based on the dilution factor and ranged from ∼10 to 2,500 or 1 to 3.4 log_10_ TCID_50_ per PCR reaction.

To examine the specificity and the ability of the array to detect different isolates within subtypes of HIV-1 and different isolates of HIV-2 by the array, we additionally tested our primers with another panel, developed by the Southern Research Institute. This panel contained three different isolates from each subtype (A to G) of HIV-1 group M, three isolates from HIV-1 group O, one isolate from HIV-1 group N and three different isolates of HIV-2. The infectious dose of each HIV isolate in the panel was determined by TCID_50_ (median tissue culture infective dose) titration and the dose of virus used in each PCR reaction was calculated. The results of testing of HIV-1 specific primers are shown in Figure 1. In this experiment we evaluated the coverage of HIV-1 variants and estimated the relative sensitivity of the four HIV-1 specific primer sets based on cycle threshold (C(t)) values obtained with each isolate tested. Two primer sets targeting the conserved *pol* regions (NP170/171 and NP175/174) were able to detect most of the HIV-1 subtypes with a high sensitivity (C(t) = 15–25). Only one primer set (NP175/174) detected both the group N isolate and all three isolates of group O with Ct = 20–35. We did not detect cross-reactivity of HIV-1 or HIV-2 specific primers with the other type of HIV. All three HIV-2 isolates studied (1–3 log_10_ TCID_50_/PCR input) were detected with all HIV-2 specific primer sets with a low Ct value: 12–20 (data not shown).

### 5. Evaluation of Sensitivity and Specificity of the Real-time PCR Viral Array with DNA Standards and Clinical Blood Samples of Patients Infected with HIV-1, HCV or HBV

After completion of sensitivity testing of primer sets with analytical panels we finalized the expected Tm peaks range for each amplicon and arranged a working array in 96-well plate format (Figure S2). To evaluate the specificity and possible cross-reactivity of primers in the array, 20–100 genome copies of each virus were spiked into 50 ng of human DNA to be used as positive templates and the same amount of human DNA was used as a negative control in each experiment. The array was tested with all targeted viruses using DNA standards (listed in Table S1). Each positive template was tested in duplicate; one human DNA negative control and one no-template control (NTC) were included in every testing. In these experiments the definition of “positive” signal was set up as the threshold cycle cut-off of C(t)≤41 (see Materials and Methods) and all Tm peaks are within the expected range (Figure S2). No cross-reactivity was detected for any primer sets in the array using DNA standards with this setting (data not shown).

**Figure 2:**
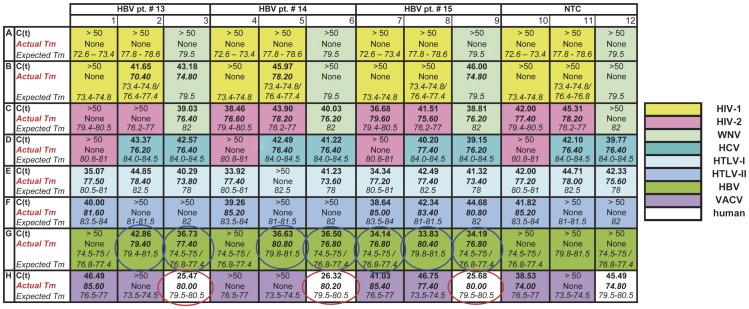
Evaluation of the real-time PCR array using blood samples derived from individuals infected with HBV. Three HBV positive patients’ samples (pt.#13–pt.#15) and one no-template control (NTC) were tested in one assay. The color scheme, shown as a footnote, refers to each virus and reflects the specific primer sets loaded into the array. Expected Tm peaks for each amplicon are shown in italic, experimentally obtained Tm peaks are shown in bold italic, and C(t) values from the experimental testing are shown in bold for each of the primer sets. The C(t) and Tm peaks that reflect the positive signals are circled: only positive signals from HBV (blue circles) and human beta-globin gene (red circles) were detected in the experiment.

Seventeen (17) clinical plasma samples (obtained from NIH Blood Bank) from donors who tested positive for HIV-1, HBV, or HCV were used to evaluate the array sensitivity and specificity. The genome copy of each virus in virus-positive plasma samples was previously determined by the NIH Blood Bank using commercial assays approved for donor screening. Representative results from the testing of three HBV-positive plasma samples (pt.#13–pt.#15) using our array are shown on Figure 2. The threshold cycle cut off of the assay performed with plasma samples was set up as C(t)≤43 (see Materials and Methods). The wells with C(t) lower than the cut-off and the obtained Tm peaks within the expected Tm range indicate positive amplification of HBV target genes and are highlighted with blue circles (Figure 2, green color code for HBV). For samples #13 and #14 Tm peaks of the products, obtained with only two HBV specific primer sets (wells G2, G3– pt.#13 and wells G5, G6–pt.#14), were within the expected range, with C(t) values of 36.7–42.9 and 36.5–36.6 respectively. For sample #15, all three HBV specific primer sets gave the Tm peaks within the expected range (wells G7, G8 and G9), and the C(t) values range was 33.8–34.2. The genome copy number of HBV in plasma samples #13–15 was 151–518 copies/ml, corresponding to 6–21 copies/PCR. The wells (white color code, red circles) with the human beta-globin gene specific primer set, serving as the internal control, produced positive signals from all three plasma samples tested, with C(t) values of 25.5–26.3 (wells H3, H6 and H9 - Figure 2), which shows that the quality of the RNA/DNA sample preparation was equally good for the plasma of these three patients.

The final Tm range for each primer set was adjusted after testing of this clinical panel. Based on the results from the testing of clinical samples and according to NSA performed during the design, two primer sets targeting the S gene region of HBV (NP11/97 and NP11/97-mod) have two different Tm ranges depending on the HBV genotype (Figure 2– wells G1 and G3). The lower Tm range (74.5–75.5°C) was detected for genotype A and the higher Tm range (76.8–77.4°C) - for genotypes B, C, and D. The difference in Tm ranges occurred due to single nucleotide polymorphism (SNP) in the amplicon sequence leading to a difference in the G/C nucleotide content of the products.

Table 4 summarizes the results from testing the 17 virus-positive clinical samples using the developed array. At least two different specific primer sets could detect a target virus in all cases, with one exception: one HIV-1 positive sample (#4) with ∼4 viral genome copies per PCR reaction was amplified with only one primer set (NP175/174). HIV-1 positive clinical samples (#1–#3) with 51–80 viral genome copies/ml of plasma or 2–3 copies/PCR reaction were detected with at least two primer sets. All six HCV-positive samples (#7 to #12) with 16–2,570 viral genome copies/ml of plasma tested positive with both HCV-specific primer sets. Three HBV-positive samples (#15, #16, #17) at 21–66 copies/PCR tested positive by the three HBV-specific primer sets and two HBV-positive samples (#13, #14) with 6–10 copies/PCR tested positive with two out of three HBV-specific primer sets. It is important to note that none of the primer sets showed any cross-reactivity with other viruses in the panel of clinical samples tested.

**Table 4:** Tm and *C(t)* values obtained with primer sets specific for HIV-1, HCV, or HBV in testing of 17 human clinical samples in the format of PCR array targeting eight different viruses.

				Specific primer sets used in the array:							
Virus/ Sample	Copies/ml^a^	V (ml)^b^	Copies/PCR^c^	NP3/4		NP51/52		NP170/171		NP175/174	
HIV-1				Tm	*C(t)*	Tm	*C(t)*	Tm	*C(t)*	Tm	*C(t)*
1	51	1	*2*	**73.0**	***35.6***	**78.6**	***46.4***	**74.4**	***37.3***	–	N/A
2	80	1	*3*	–	N/A	–	N/A	**74.4**	***42.3***	**73.8/77.4**	***41.3***
3	80	1	*3*	**72.6**	***38.8***	–	N/A	–	N/A	**73.4/76.0**	***42.2***
4	106	1	*4*	–	N/A	–	N/A	–	N/A	**74.0/76.8**	***39.8***
5	9,094	0.5	*190*	**73.4**	***34.9***	**77.8**	***39.3***	**75.2**	***34.0***	**74.0/77.4**	***38.9***
6	89,109	0.5	*1,857*	**73.4**	***30.0***	**78.0**	***33.8***	**74.8**	***31.1***	**73.0/77.8**	***36.1***
				**NP13/14**		**NP13-mod/14**		**N/A**		**N/A**	
**HCV**				**Tm**	***C(t)***	**Tm**	***C(t)***				
7	786	0.5	*16*	**84.2**	***37.6***	**84.0**	***35.3***				
8	2,352	0.5	*49*	**84.0**	***32.9***	**84.0**	***32.6***				
9	3,051	0.5	*64*	**83.8**	***34.1***	**83.8**	***33.7***				
10	11,502	0.5	*240*	**84.2**	***28.9***	**84.2**	***28.3***				
11	49,140	0.5	*1,024*	**84.2**	***27.8***	**84.2**	***27.3***				
12	123,390	0.5	*2,570*	**84.6**	***29.2***	**84.4**	***28.9***				
				**NP11/97**		**NP94/100**		**NP11/97-mod**		**N/A**	
**HBV**				**Tm**	***C(t)***	**Tm**	***C(t)***	**Tm**	***C(t)***		
13	151	1	*6*	–	N/A	**79.4**	***42.9***	**77.4**	***36.7***		
14	244	1	*10*	–	N/A	**80.8**	***36.6***	**76.8**	***36.5***		
15	518	1	*21*	**76.8**	***34.1***	**80.4**	***33.8***	**76.8**	***34.2***		
16	3,248	0.5	*67*	**77.2**	***34.3***	**79.6**	***36.0***	**77.0**	***35.0***		
17	15,889	0.1	*66*	**75.2**	***36.8***	**81.0**	***35.7***	**75.2**	***36.4***		

a - number of viral genome copies per ml of plasma, estimated at NIH Blood Bank;

b – V – volume of the sample analyzed;

c - genome copies per PCR, estimated based on the number of copies per ml, considering that each sample was tested in the format of PCR array with 24 different primer sets;

“-” – no specific Tm peak was detected; N/A – non applicable. Each clinical sample was tested once (no PCR repeats were used) due to limitation of material.

## Discussion

In the study presented here we applied a real-time PCR array approach in a 96-well plate platform for detection of a group of target viral pathogens. In contrast to TaqMan PCR, which is commonly used for viral diagnostics, this platform based on PCR with SYBR Green chemistry supports simultaneous detection and identification of 24 different targets corresponding to eight different viruses. PCR based on SYBR Green staining of the double stranded DNA is economically affordable and allows for the detection of mutants with SNPs within the amplicon sequence [34]. The strategy of primer selection for the array included the sequential use of different bioinformatics programs to identify highly-specific primer sets with a maximal variants’ coverage of the targeted viruses, while working under “universal” PCR conditions. Experimental selection process using a panel of DNA or RNA analytical standards allowed choosing two to four most sensitive and specific primer sets for each targeted virus. The sensitivity (in copy number per PCR reaction) and the intra and inter-assay reproducibility of quantification were characterized for each primer set selected for the array.

FDA/CBER analytical plasma panels were used to assess the detection sensitivity of the primer sets selected for the working array. The HIV-2 and HCV RNA was detected at as low as 10 genome copies/ml of human plasma by one out of three and two out two primer sets, respectively. Similarly, WNV RNA and HBV DNA were detected at 50 genome copies/ml of plasma by two out of three and one out of three selected primer sets respectively.

All four of the selected HIV-1 specific primer sets detected group M, subtype B, the most common subtype of HIV-1 in the Americas and Western Europe [35], at 50–00 genome copies/ml of plasma. However, the array was able to amplify all other subtypes, excluding subtype F, of HIV-1 group M with only one primer set (NP170/171), targeting the conserved *pol* region, at 100–1,000 genome copies/ml of plasma. In addition, the array amplified the group N isolate at 1,000 copies/ml with another primer set (NP175/174) targeting the conserved *pol* region. All the selected HIV-1 specific primer sets of the array failed to amplify the group O isolate (detection limit is >1,000 copies/ml of plasma). In comparison, the limit of detection (LOD) of recently improved commercial multiplex NAT assays for the broad spectrum of HIV-1 group M isolates is 40 copies/ml, and the LOD for group O is 200 copies/ml [36]–[38]. Thus, specific primer sets targeting group O isolates of HIV-1, as well as subtype F (group M) will need to be included in the array to increase the coverage of all existing subtypes/groups of the HIV-1. No cross-reactivity was shown for HIV-1 or HIV-2 specific primers with the other type of HIV in a testing with medically relevant levels of viruses in a diversity panel containing 25 different HIV-1 isolates and three natural HIV-2 isolates collected worldwide.

There is presently no US Food and Drug Administration (FDA) approved PCR-based NAT testing for blood donors’ screening for HTLV-1 and HTLV-2. There is no official FDA (or World Health Organization (WHO)) viral panel released for these two viruses. It is also difficult to obtain HTLV-1 or HTLV-2 positive blood donors’ samples in the United States. In the absence of the analytical panels and clinical samples we tested HTLV-1, HTLV-2 and VACV specific primers only with cell culture derived DNA and with DNA standards. The minimum detection limit of HTLV-1 and HTLV-2 specific primer sets estimated with analytical DNA standards was 5–10 genome copies/PCR reaction, which is in the same range as for other primers included to the array. The coverage of the viral variants for the primers targeting these viruses was estimated *in silico* using multiple sequence alignments performed with complete genome sequences available in Gen Bank.

The working array arranged in 96-well plate format was subsequently tested for specificity and potential cross-reactivity with human DNA and with each of the targeted viruses. None of the primer sets selected for a particular target virus in the array produced non-specific cross-reaction toward the other viruses. Comparative performance of the array was also evaluated through the testing of 17 clinical specimens from the United States patient population. All 17 samples were correctly identified in our PCR array with a high sensitivity to contain HIV-1, HCV, or HBV. We found that a combination of several primer sets targeting each virus in the array allows for the detection of different variants of the virus; however, it makes the absolute quantification with uniform DNA/RNA standards a challenge. Quantification by the assay is not always possible when the genetic group of the viral isolate being tested is different than the assay standards. This is one of the reasons why in certain cases the commercial assays underestimate viral loads by up to 1–10 log_10_ copies per ml [37]–[39]. Nevertheless, relative quantification can be done using all primer sets selected for the array, and the absolute quantification can be performed with DNA/RNA standards using the primers targeting the most conserved genome areas.

There are several commercial qualitative multiplex NAT assays now available on the market simultaneously targeting three most important blood-borne viral pathogens (HIV-1, HCV and HBV) [40]. One of them was recently approved by U.S. FDA for screening of blood and organ donors (http://www.fda.gov/BiologicsBloodVaccines/BloodBloodProducts/ApprovedProducts/LicensedProductsBLAs/BloodDonorScreening/InfectiousDisease/ucm306073.htm).

We compared the LOD of these multiplex NAT assays [40] with the results of our working PCR array in sensitivity of testing against these important viruses. The LOD for HBV are 38.1–195 geq/ml by “Procleix Ultrio Tigris” and 9.2–37.5 geq/ml by “Cobas s 201”. The LOD for HCV are 15.3–32.6 geq/ml by “Procleix Ultrio Tigris” and 26.0–81.0 geq/ml by “Cobas s 201”. The sensitivity of detecting HBV and HCV by our PCR array appeared to be in a similar range. The LOD for HIV-2 (subtype A) is 43.4 geq/ml by “Cobas s 201”. Our array has a comparable sensitivity in detecting this virus. However, as described above, the sensitivity of our PCR array in the detection of the broader spectrum of different subtypes of HIV-1 isolates (50–1,000 geq/ml) would need to be improved. The LOD for HIV-1 are 17.5–48.2 geq/ml by “Procleix Ultrio Tigris”, 34.7–5,187 geq/ml by “Cobas s 201.

Other PCR-coupled techniques have been developed previously for highly-sensitive pathogens’ detection that could reach the sensitivity of the assay up to 1 genome copy per PCR reaction. For example the bioactive amplification with probing (BAP), utilizing a nested PCR and magnetic bead-based hybridization with the specific probe, has been developed for the detection of bovine and avian viruses [41]–[43]. In spite of the exceeding sensitivity of such assays targeting a single virus, it may be difficult to adapt the approach or method to meet the main objective of simultaneous detection of multiple target viral pathogens by an array using universal PCR conditions.

It is important to note that this array was developed to be adapted by any laboratory. Comparison of experimentally obtained Tm peaks to the range of expected specific Tm peaks allows rapid identification or exclusion of the viral pathogen in a sample. This is an initial study to examine the suitability of using PCR arrays for the detection of a group of target viruses. The current array was developed utilizing five previously published and 19 originally designed primers sets. However, it can be expanded to a larger number of targets for the same virus. Targeting of several genome areas increases the detection sensitivity of the target virus and provides an intra-assay confirmation of positive signals. Additionally, any new virus of interest can be added to the list of targeted pathogens. Efforts are underway to test the utility of this real-time PCR array using samples with more diverse biological origin and pathogen content.

## Supporting Information

Figure S1 **Standard and melting curves generated for HIV-1 specific primer set NP3/4 using analytical DNA standards.** Serial dilutions of HIV-1 DNA standards (5 to 10^4^ genome copies, shown in red) spiked into 50 ng of human DNA were run in four experimental repeats each. A. Amplification curve. B. Standard curve. C. Melting curve. HIV-1 positive cells (20 ng of total DNA) are shown in black, no-template control (NTC) – in light blue, “negative” human DNA control – in green. The limit of sensitivity of this primer set determined in the experiment was 10 genome copies/PCR.
(TIF)

Figure S2 **Real-time PCR viral array design.** Primer names (shown in bold), melting peaks of melting temperature (Tm) (bold italic) and PCR product size (underlined) are indicated in each well of 96-well plate image. The color scheme, shown on the right, refers to each virus.
(TIF)

Table S1 **List of the synthetic templates used as analytical standards in real-time PCR assay.**
(DOC)

Table S2 **Intra and inter-assay reproducibility of viral quantification evaluated using analytical DNA or RNA standards for each virus specific primer set selected for the real-time PCR array.**
(DOC)
