# Supplementary figures and images for: Development of Real-Time PCR Array for Simultaneous Detection of Eight Human Blood-Borne Viral Pathogens
Source: PLoS One. 2012 Aug 17;7(8):e43246. doi: 10.1371/journal.pone.0043246 (PMC3422334; doi:10.1371/journal.pone.0043246)

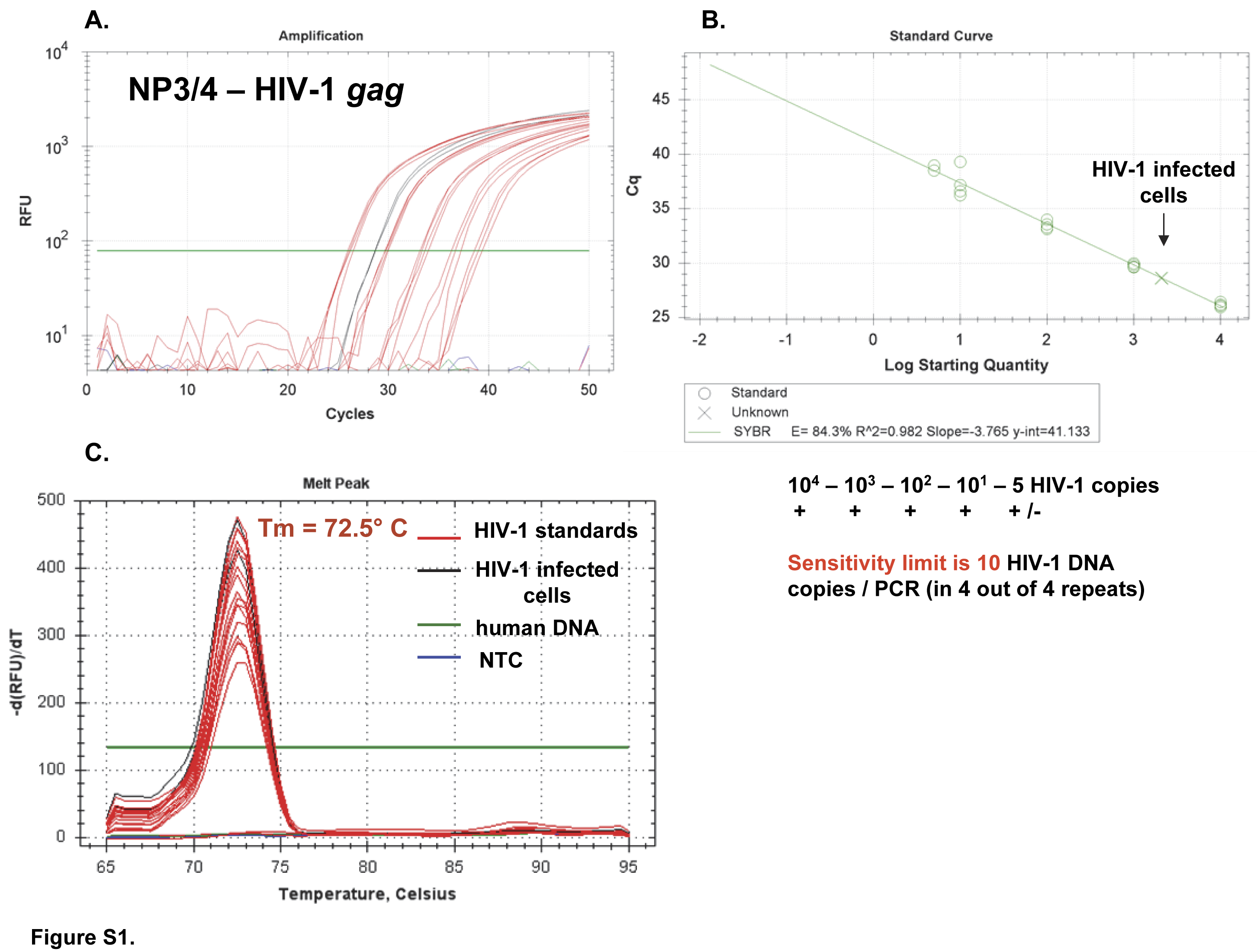

Supplement: Figure S1 — Standard and melting curves generated for HIV-1 specific primer set NP3/4 using analytical DNA standards. Serial dilutions of HIV-1 DNA standards (5 to 104 genome copies, shown in red) spiked into 50 ng of human DNA were run in four experimental repeats each. A. Amplification curve. B. Standard curve. C. Melting curve. HIV-1 positive cells (20 ng of total DNA) are shown in black, no-template control (NTC) – in light blue, “negative” human DNA control – in green. The limit of sensitivity of this primer set determined in the experiment was 10 genome copies/PCR. (TIF) [file pone.0043246.s001.tif]

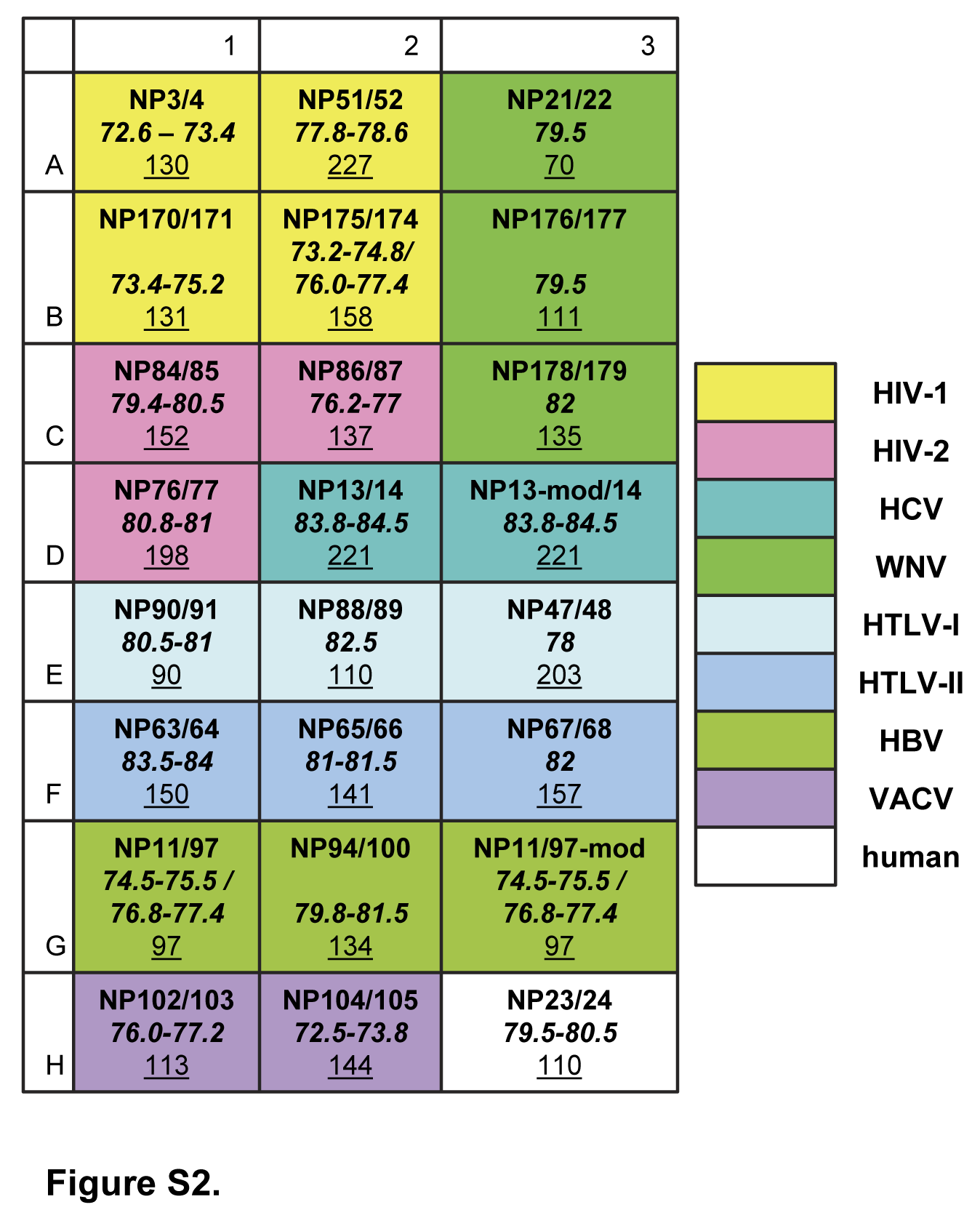

Supplement: Figure S2 — Real-time PCR viral array design. Primer names (shown in bold), melting peaks of melting temperature (Tm) (bold italic) and PCR product size (underlined) are indicated in each well of 96-well plate image. The color scheme, shown on the right, refers to each virus. (TIF) [file pone.0043246.s002.tif]
