# Supplementary material for: Development of Real-Time PCR Array for Simultaneous Detection of Eight Human Blood-Borne Viral Pathogens
Source: PLoS One. 2012 Aug 17;7(8):e43246. doi: 10.1371/journal.pone.0043246 (PMC3422334; doi:10.1371/journal.pone.0043246)
Supplement: Table S1 — List of the synthetic templates used as analytical standards in real-time PCR assay. (DOC) [file pone.0043246.s003.doc]

**Table S1.** **List of the synthetic templates used as analytical standards in real-time PCR assay.**

| Virus | Plasmid name ^a^ | Gene | Location (nucleotide numbers ^b^) | Source of template DNA or RNA |
| --- | --- | --- | --- | --- |
| HTLV-1 | pNP1 | LTR, *gag, pro, pol* | 45-3132 | C91/PL cells |
| HTLV-1 | pNP6 | *pol, env* | 3100-5793 | C91/PL cells |
| HTLV-1 | pNP8 | *pol* | 3100-3208 | C91/PL cells |
| HTLV-2 | pNP2 | *pol, env* | 3745-6105 | 81-F-3 cells |
| HIV-1 | pNP3 | *gag, pol* | 1090-2910 | H9/IIIB cells |
| HIV-1 | pNP13 | *pol* | 4365-4523 | H9/IIIB cells |
| HIV-2 | pNP4 | *gag* | 1467-2159 | 190/HIV-2 cells |
| HIV-2 | pNP5 | *env* | 8988-9186 | 190/HIV-2 cells |
| HBV | pNP7 | S gene | 379-1602 | Genotype A; human plasma |
| HBV | pRW1 | core | 1805-2458 | Genotype A; human plasma |
| HCV | pRW2 | 5’NTR, C (capsid) | 1 - 900 | Genotype 1a; human plasma |
| VACV | pNP11 | Haemagglutinin | 123056-123069 | HeLa S3 cells/ strain WR |
| VACV | pNP12 | B5R gene (membrane glycoprotein) | 168537-168681 | HeLa S3 cells/ strain WR |
| WNV | pNP14 | E protein | 1160-1229 | HU2002 strain of WNV |
| WNV | pNP15 | NS5 protein | 8147-8258 | HU2002 strain of WNV |
| WNV | Chimera  WNV/DEN4 | prM - E ^1^ | 403-2400 | WNV parental strain is NY99 [1]. |
| Human | pNP10 | Beta-globin | chr. 11,  11p15.5  (110 b.p.) | CEM cells |

^a^ - all pNP and pRW plasmids were created by TA-cloning into pGEM-T-Easy vector (Promega) which contain SP6/T7 promoters for *in vitro* transcription. *In vitro* transcribed RNA standards were used for RNA viruses; ^b^ – nucleotide numbers are according to sequences available in GenBank (see Materials and Methods for accession numbers); ^1^ – prM –E (pre-membrane and envelop) genes of WNV in the backbone of Dengue type 4.

**References:**

1. Pletnev AG, Putnak R, Speicher J, Wagar EJ, Vaughn DW (2002) West Nile virus/dengue type 4 virus chimeras that are reduced in neurovirulence and peripheral virulence without loss of immunogenicity or protective efficacy. Proc Natl Acad Sci U S A 99: 3036-41.
