# Supplementary material for: Development of Real-Time PCR Array for Simultaneous Detection of Eight Human Blood-Borne Viral Pathogens
Source: PLoS One. 2012 Aug 17;7(8):e43246. doi: 10.1371/journal.pone.0043246 (PMC3422334; doi:10.1371/journal.pone.0043246)
Supplement: Table S2 — Intra and inter-assay reproducibility of viral quantification evaluated using analytical DNA or RNA standards for each virus specific primer set selected for the real-time PCR array. (DOC) [file pone.0043246.s004.doc]

**Table S2. Intra and inter-assay reproducibility of viral quantification evaluated using analytical DNA or RNA standards for each virus specific primer set selected for the real-time PCR array.**

|  |  |  | **Intra-assay C(t) values ^a^** | | | **Inter-assay C(t) values ^b^** | | |
| --- | --- | --- | --- | --- | --- | --- | --- | --- |
| **Virus** | **Primer set** | **Copy number** | **Mean** | **SD** | **CV (%)** | **Mean** | **SD** | **CV (%)** |
| **HIV-1** | **NP3/4** | 10 ^4^ | 26.233 | 0.186 | 0.708 | 26.162 | 0.207 | 0.793 |
| **(subtype B)** |  | 10 ^3^ | 29.713 | 0.112 | 0.375 | 29.545 | 0.962 | 3.256 |
|  |  | 10 ^2^ | 33.33 | 0.219 | 0.658 | 33.005 | 1.963 | 5.948 |
|  |  | 10 | 36.673 | 0.474 | 1.293 | 38.325 | 2.247 | 5.863 |
|  | **NP51/52** | 10 ^4^ | 27.453 | 0.068 | 0.247 | 27.495 | 0.100 | 0.364 |
|  |  | 10 ^3^ | 31.077 | 0.336 | 1.079 | 30.698 | 0.787 | 2.566 |
|  |  | 10 ^2^ | 33.065 | 0.021 | 0.064 | 34.217 | 0.855 | 2.499 |
|  |  | 10 | 38.14 | 1.066 | 2.793 | 38.17 | 1.050 | 2.752 |
|  |  | 5 | 40.265 | 0.120 | 0.298 | 39.536 | 1.0134 | 2.563 |
|  | **NP170/171** | 10 ^4^ | 32.16 | 0.014 | 0.043 | 32.36 | 0.512 | 1.583 |
|  |  | 10 ^3^ | 34.55 | 0.325 | 0.941 | 34.907 | 0.532 | 1.525 |
|  |  | 10 ^2^ | 37.95 | 0.127 | 0.335 | 37.584 | 0.744 | 1.981 |
|  |  | 10 | 38.885 | 1.181 | 3.036 | 39.36 | 1.168 | 2.969 |
|  | **NP175/174** | 10 ^4^ | 29.245 | 0.078 | 0.265 | 29.47 | 0.393 | 1.335 |
|  |  | 10 ^3^ | 34.53 | 0.477 | 1.381 | 34.334 | 1.837 | 5.350 |
|  |  | 10 ^2^ | 39.183 | 0.854 | 2.178 | 38.101 | 2.491 | 6.538 |
|  |  | 10 | 40.56 | 0.608 | 1.499 | 41.512 | 1.031 | 2.485 |
| **HIV-2** | **NP84/85** | 10 ^4^ | 24.097 | 0.362 | 1.501 | 24.185 | 0.344 | 1.422 |
|  |  | 10 ^3^ | 28.803 | 0.472 | 1.640 | 28.595 | 0.567 | 1.985 |
|  |  | 10 ^2^ | 32.603 | 0.618 | 1.895 | 32.53 | 0.525 | 1.615 |
|  |  | 10 | 35.66 | 0.155 | 0.436 | 35.536 | 0.240 | 0.676 |
|  | **NP86/87** | 10 ^4^ | 24.61 | 0.366 | 1.489 | 24.732 | 0.386 | 1.563 |
|  |  | 10 ^3^ | 29.047 | 0.424 | 1.462 | 29.055 | 0.347 | 1.195 |
|  |  | 10 ^2^ | 33.237 | 0.488 | 1.468 | 32.802 | 0.955 | 2.912 |
|  |  | 10 | 37.883 | 0.399 | 1.054 | 37.507 | 0.819 | 2.184 |
|  |  | 5 | 40.193 | 0.310 | 0.771 | 40.02 | 0.429 | 1.072 |
|  | **NP76/77** | 10 ^4^ | 26.41 | 0.042 | 0.160 | 26.387 | 0.100 | 0.381 |
|  |  | 10 ^3^ | 30.565 | 0.657 | 2.151 | 30.302 | 0.619 | 2.043 |
|  |  | 10 ^2^ | 33.245 | 0.077 | 0.233 | 33.425 | 0.219 | 0.655 |
|  |  | 10 | 37.65 | 0.297 | 0.788 | 37.642 | 2.972 | 7.897 |
|  |  | 5 | 37.775 | 0.360 | 0.954 | 38.262 | 0.633 | 1.656 |
| **HTLV-1** | **NP88/89** | 10 ^4^ | 25.215 | 0.233 | 0.925 | 25.45 | 0.312 | 1.22 |
|  |  | 10 ^3^ | 28.695 | 0.572 | 1.996 | 29.025 | 0.555 | 1.914 |
|  |  | 10 ^2^ | 33.7 | 0.551 | 1.636 | 33.765 | 0.327 | 0.970 |
|  |  | 10 | 37.025 | 0.261 | 0.706 | 37.153 | 0.289 | 0.778 |
|  |  | 5 | 38.9 | 0.848 | 2.181 | 38.795 | 0.703 | 1.812 |
|  | **NP47/48** | 10 ^4^ | 32.41 | 0.099 | 0.305 | 32.177 | 0.298 | 0.929 |
|  |  | 10 ^3^ | 35.875 | 0.035 | 0.098 | 36.002 | 0.148 | 0.413 |
|  |  | 10 ^2^ | 38.495 | 0.261 | 0.679 | 38.825 | 0.483 | 1.246 |
|  |  | 10 | 39.93 | 0.113 | 0.283 | 40.187 | 0.791 | 1.969 |
|  | **NP90/91** | 10 ^4^ | 26.165 | 0.091 | 0.351 | 26.26 | 0.132 | 0.506 |
|  |  | 10 ^3^ | 29.945 | 0.360 | 1.204 | 29.655 | 0.724 | 2.444 |
|  |  | 10 ^2^ | 34.13 | 0.693 | 2.030 | 34.2 | 0.409 | 1.197 |
|  |  | 10 | 36.51 | 0.424 | 1.162 | 36.805 | 0.521 | 1.417 |
| **HTLV-2** | **NP63/64** | 10 ^4^ | 25.215 | 0.502 | 1.991 | 25.072 | 0.965 | 3.851 |
|  |  | 10 ^3^ | 29.345 | 0.332 | 1.132 | 29.215 | 0.464 | 1.591 |
|  |  | 10 ^2^ | 33.935 | 0.615 | 1.812 | 32.922 | 1.782 | 5.415 |
|  |  | 10 | 38.16 | 0.099 | 0.259 | 37.716 | 0.771 | 2.044 |
|  |  | 5 | 38.33 | 0.240 | 0.627 | 38.865 | 0.708 | 1.823 |
|  | **NP65/66** | 10 ^4^ | 24.73 | 0.551 | 2.230 | 24.722 | 0.409 | 1.656 |
|  |  | 10 ^3^ | 29.795 | 0.035 | 0.118 | 29.702 | 0.224 | 0.754 |
|  |  | 10 ^2^ | 33.35 | 0.480 | 1.441 | 33.047 | 1.117 | 3.381 |
|  |  | 10 | 36.365 | 0.289 | 0.797 | 35.54 | 1.443 | 4.061 |
|  | **NP67/68** | 10 ^4^ | 27.827 | 0.232 | 0.836 | 27.642 | 0.488 | 1.768 |
|  |  | 10 ^3^ | 30.756 | 0.148 | 0.481 | 31.259 | 1.744 | 5.588 |
|  |  | 10 ^2^ | 36.24 | 0.068 | 0.187 | 36.422 | 0.642 | 1.765 |
|  |  | 10 | 37.597 | 0.631 | 1.678 | 36.628 | 1.830 | 4.99 |
| **HBV** | **NP11/97** | 10 ^4^ | 29.6 | 0.466 | 1.576 | 29.807 | 0.360 | 1.209 |
|  |  | 10 ^3^ | 34.065 | 0.106 | 0.311 | 34.017 | 0.205 | 0.602 |
|  |  | 10 ^2^ | 35.97 | 0.183 | 0.511 | 35.807 | 0.777 | 2.171 |
|  |  | 10 | 36.947 | 0.968 | 2.620 | 36.52 | 1.428 | 3.911 |
|  | **NP94/100** | 10 ^4^ | 24.15 | 0.113 | 0.468 | 24.262 | 0.198 | 0.816 |
|  |  | 10 ^3^ | 27.36 | 0.396 | 1.447 | 27.33 | 0.257 | 0.943 |
|  |  | 10 ^2^ | 30.55 | 0.056 | 0.185 | 30.565 | 0.135 | 0.444 |
|  |  | 10 | 33.805 | 0.779 | 2.305 | 35.97 | 1.131 | 3.145 |
|  | **NP11/97-mod** | 10 ^4^ | 29.65 | 0.495 | 1.669 | 30.087 | 0.698 | 2.320 |
|  |  | 10 ^3^ | 34.25 | 0.353 | 1.032 | 34.175 | 0.275 | 0.805 |
|  |  | 10 ^2^ | 36.1 | 0.282 | 0.783 | 35.95 | 0.772 | 2.148 |
|  |  | 10 | 37.03 | 0.984 | 2.658 | 36.7 | 1.555 | 4.238 |
| **HCV** | **NP13/14** | 10 ^3^ | 27.81 | 0.070 | 0.254 | 26.62 | 0.829 | 3.114 |
|  |  | 10 ^2^ | 31.755 | 0.417 | 1.313 | 30.597 | 1.358 | 4.439 |
|  |  | 10 | 34.945 | 0.091 | 0.263 | 34.177 | 0.889 | 2.601 |
|  |  | 5 | 36.555 | 0.063 | 0.174 | 36.407 | 0.222 | 0.611 |
|  | **NP13-mod/14** | 10 ^3^ | 27.145 | 0.063 | 0.234 | 25.46 | 0.650 | 2.55 |
|  |  | 10 ^2^ | 30.76 | 0.155 | 0.505 | 30.157 | 0.701 | 2.327 |
|  |  | 10 | 34.77 | 0.169 | 0.488 | 34.022 | 0.871 | 2.561 |
|  |  | 5 | 35.73 | 0.424 | 1.187 | 35.885 | 0.324 | 0.903 |
| **WNV** | **NP21/22** | 10 ^4^ | 23.815 | 0.261 | 1.098 | 23.907 | 0.448 | 1.874 |
|  |  | 10 ^3^ | 27.742 | 0.478 | 1.724 | 27.52 | 0.693 | 2.518 |
|  |  | 10 ^2^ | 31.145 | 0.063 | 0.204 | 31.047 | 0.125 | 0.403 |
|  |  | 10 | 33.93 | 0.622 | 1.833 | 33.99 | 0.575 | 1.704 |
|  | **NP176/177** | 10 ^4^ | 26.415 | 0.544 | 2.061 | 26.08 | 0.792 | 3.039 |
|  |  | 10 ^3^ | 29.82 | 0.028 | 0.094 | 29.166 | 0.530 | 1.817 |
|  |  | 10 ^2^ | 32.2 | 0.135 | 0.420 | 31.953 | 1.066 | 3.337 |
|  |  | 10 | 34.995 | 0.360 | 1.03 | 35.223 | 0.322 | 0.914 |
|  | **NP178/179** | 10 ^4^ | 25.1 | 0.141 | 0.563 | 24.975 | 0.170 | 0.683 |
|  |  | 10 ^3^ | 29.06 | 0.014 | 0.048 | 29.157 | 0.128 | 0.440 |
|  |  | 10 ^2^ | 32.28 | 0.183 | 0.569 | 32.12 | 0.330 | 1.030 |
|  |  | 10 | 34.175 | 0.205 | 0.600 | 34.372 | 0.597 | 1.736 |
| **Vaccinia** | **NP102/103** | 10 ^4^ | 23.16 | 0.622 | 2.686 | 23.187 | 0.382 | 1.648 |
|  |  | 10 ^3^ | 25.575 | 0.778 | 3.042 | 26.197 | 1.260 | 4.810 |
|  |  | 10 ^2^ | 30.495 | 1.011 | 3.315 | 30.415 | 0.608 | 1.999 |
|  |  | 10 | 33.745 | 0.898 | 2.661 | 34.585 | 1.264 | 3.657 |
|  |  | 5 | 35.89 | 0.367 | 1.024 | 37.326 | 2.501 | 6.702 |
|  | **NP104/105** | 10 ^4^ | 24.193 | 0.228 | 0.944 | 24.025 | 0.384 | 1.602 |
|  |  | 10 ^3^ | 27.893 | 0.235 | 0.844 | 27.885 | 0.192 | 0.691 |
|  |  | 10 ^2^ | 31.867 | 0.812 | 2.550 | 32.01 | 0.932 | 2.911 |
|  |  | 10 | 38.1 | 0.579 | 1.521 | 35.757 | 1.359 | 3.801 |
|  |  | 5 | 38.75 | 0.670 | 1.731 | 38.75 | 0.670 | 1.731 |

**^a^** - three replicates of each dilution were run in one assay; ^b^ – three separate assays were run consisted of three replicates each.
